# A Study of the Clinical Profile and Management of Children With Anorectal Malformations

**DOI:** 10.7759/cureus.36772

**Published:** 2023-03-27

**Authors:** Roshan Chanchlani, K S Budhwani

**Affiliations:** 1 Paediatric Surgery, All India Institute of Medical Sciences (AIIMS) Bhopal, Bhopal, IND; 2 Paediatric Surgery, Gandhi Medical College, Bhopal, IND

**Keywords:** paediatric surgery, neonate, colostomy, anterior sagittal anorectoplasty, anorectal malformations

## Abstract

Introduction

The diagnosis of anorectal malformations (ARMs) antenatally is rare, and most of these cases are diagnosed in the neonatal period. The defects range from easily treated minor anomalies with good prognosis to those that are difficult and complex. The associated anomalies in these malformations are important determinants for the prognosis and outcome of these cases.

Material and methods

The present study was carried out in the department of pediatric surgery in a tertiary care institute. Patients presenting with ARMs in the neonatal period, those reported for definitive surgery, and patients for colostomy closure surgery were included. Patients who died before surgical treatment were excluded from the study. Convenience sampling of 100 patients who met the inclusion criteria was performed until the sample size was reached.

Results

Out of 63 cases of high variety, 84.12% were males and 15.87% were females. Out of 37 patients of low variety, 43.24% were males and 56.75% were females. The anomalies of the urogenital system were present in 22 (34.92%) out of 63 cases of high ARMs and 10 (27.02%) out of 37 cases of low ARMs. In the male patients, anocutaneous fistula was in 16 (23.18%) of cases. Out of 31 females in the present study, anorectal agenesis with anovestibular fistula was seen in 19 (61.29%) cases. In the early complications, significant bleeding and urethral injury were seen in one (2.63%) patient each. However, among the late complications, anal stenosis, mucosal prolapse, and wound infection were seen in seven (18.42%), five (13.15%), and four (10.52%) patients, respectively.

Conclusion

A holistic approach to the management of ARMs is needed with a long-term goal of achieving urinary and fecal continence with good quality of life. The outcome of surgery is dependent on the specific type of malformation, but the results are better when intervention is done early.

## Introduction

Congenital malformations of the anus and rectum are fairly common and frequently encountered in neonatal surgical emergencies. The crux of proper management and future results mainly depends upon the exact diagnosis of the type of anomaly, identification of associated fistulous communications, and the presence of other congenital anomalies [1-3]. The National Institutes of Health, United States Department of Health, and Human Services stated that about one in every 5,000 neonates is born with anorectal malformations (ARMs), with an increased preponderance among males [4-6]. Various surgical procedures have been adopted depending on the level of the distal rectal pouch and its relation to the puborectalis sling. In high anomaly, a preliminary colostomy followed by a definite procedure like posterior sagittal anorectoplasty (PSARP) or pull-through procedure at a suitable time and later the closure of colostomy has been the gold standard for over 150 years, whereas low-type ARMs are treated by a definite perineal minor surgical procedure at birth [5].

Recently, primary pull-through operations without colostomy using the posterior sagittal approach in newborns with high and intermediate ARMs are being done, to avoid high morbidity and discomforts associated with staged procedures [4]. For surgical repair of a variety of ARM cutbacks, anoplasty or anal transposition has been used. For female children having an intermediate and low type of anomaly, anterior sagittal anorectoplasty (ASARP) is done. 

Most of the patients with high ARMs were managed in stages with initial diversion procedures in the form of colostomy and definite procedures such as posterior sagittal anorectoplasty and abdominal perineal pull-through. The posterior sagittal approach gives good exposure of the area so as to facilitate meticulous repair of different fistulae, and maximum utilization of the striated muscles resulted in better continence near the normal anus. However, the abdominal perineal approach is required when the rectal pouch and fistula are high [3,4].

Patients with low ARMs were managed by anoplasty in both males and females and anterior sagittal anorectoplasty in females [5]. Anterior sagittal anorectoplasty is a suitable technique for the management of vestibular fistulae in females as proper mobilization of the rectum and placement of the rectum in the sphincter complex gives good continence [5]. The present study was conducted with the aim to find the incidence of various types of ARMs and associated anomalies and to emphasize the importance of managing these anomalies timely so as to minimize the overall morbidity and mortality in children suffering from ARM.

## Materials and methods

This cross-sectional study was carried out in the department of pediatric surgery for the period of three years from 2005-2008 in a tertiary care institute (Government Medical College, Bhopal) after a written consent form and clearance from the Institutional Review Board with vide letter number "Government Medical College/2426/MC/08". The study included all patients presenting with ARMs in the neonatal period, those reported for definitive surgery, and colostomy closure. Patients who died before surgical treatment were excluded from the study. A total of 100 patients were studied in the period of three years. Recruitment of patients was done in the emergency department, neonatal ward, and pediatric medical and surgical wards and thereafter followed up at the pediatric surgical outpatient department.

The diagnosis of ARMs was made using a combination of clinical and radiological assessments. Abdominal ultrasound was done to detect any other abdominal pathology associated with ARMs. Echocardiography was done in all patients to rule out VACTERAL associations. Among patients with congenital pouch colon (CPC), incomplete/partial type of CPC was commonly seen, and most of the babies with CPC had genitourinary (colovesical fistula). These patients were managed by abdominal perineal pull-through with excision of the pouch colon and ligation of the colovesical fistula. All the patients initially underwent diversion procedures [1,2]. The data were presented in a Microsoft Excel sheet as numbers (percentage).

## Results

All patients were successfully enrolled in the study. Out of 63 cases of high variety, 53 were males (84.12%) and 10 were females (15.87%). Out of 37 patients of low variety, 16 were males (43.24%) and 21 were females (56.75%). Of the 100 cases of ARM, 69 (69%) were male and 31 (31%) were female (Table 1). 

**Table 1:** Type of Anomaly and Sex Distribution in Relation to the Type of Anomaly

Type	Male		Female		Total
	Number	Percentage	Number	Percentage	Number (Percentage)
High	53	84.12	10	15.87	63 (63)
Low	16	43.24	21	56.75	37 (37)

Table 2 depicts the associated anomalies with the urogenital system. The anomalies of the urogenital system were present in 22 (34.92%) out of 63 cases of high ARMs and 10 (27.02%) out of 37 cases of low ARMs. In the urological anomalies, the most common anomaly was vesicoureteric reflux. Other anomalies in high variety are renal agenesis, multicystic kidney, horseshoe kidney, hypoplastic kidney, and duplex kidneys. In the genital system, undescended testis and hypospadias were most commonly seen in four cases. In high variety, three and two cases of ventricular septal defects and atrial septal defects were found, respectively. However, in low variety, two cases had ventricular septal defects. Out of all, 10 cases of sacral vertebral anomalies were found composed of seven (7%) patients of high variety and 3 (3%) of low type. In the gastrointestinal system, six cases of the anomaly were found which included 5 (5%) and 1 (1%) of tracheoesophageal fistula and duodenal atresia, respectively. Other anomalies like cleft lip/palate polydactyly (one case), radial aplasia, and club foot were also detected (Table 2).

**Table 2:** Type of Associated Anomalies

Anomalies		High	Low	Male	Female
External Defects	Cleft Lip/Palate	1	0	1	0
	Polydactyly	1	0	1	0
	Total	2	0	2	0
Cardiovascular System	Ventricular Septal Defect	3	2	4	1
	Atrial Septal Defect	2	0	2	0
	Total	5	2	6	1
Genital System	Undescended Testis	3	1	4	0
	Unicornuate Uterus	1	0	0	1
	Bifid Scrotum	2	2	4	0
	Hypospadias	2	2	4	0
	Megalourethra	0	1	1	0
	Total	8	6	13	1
Urinary System	Vesico-ureteric Reflux	10	3	11	2
	Renal Agenesis	1	0	1	0
	Hypoplastic Kidney	1	0	1	0
	Multicystic Kidney	0	1	0	1
	Horseshoe Kidney	1	0	0	1
	Duplex Renal	1	0	1	0
	Collecting System	0	0	0	0
	Total	14	4	14	4
Vertebra	Sacral Anomalies	7	3	8	2
	Radial Aplasia	1	0	1	0
	Clubfoot	1	0	0	1
	Total	9	3	9	3
Gastrointestinal System	Tracheo-oesophageal	2	3	1	4
	Fistula	0	1	0	1
	Duodenal Atresia	0	0	0	0
	Total	2	4	1	5

**Figure 1:**
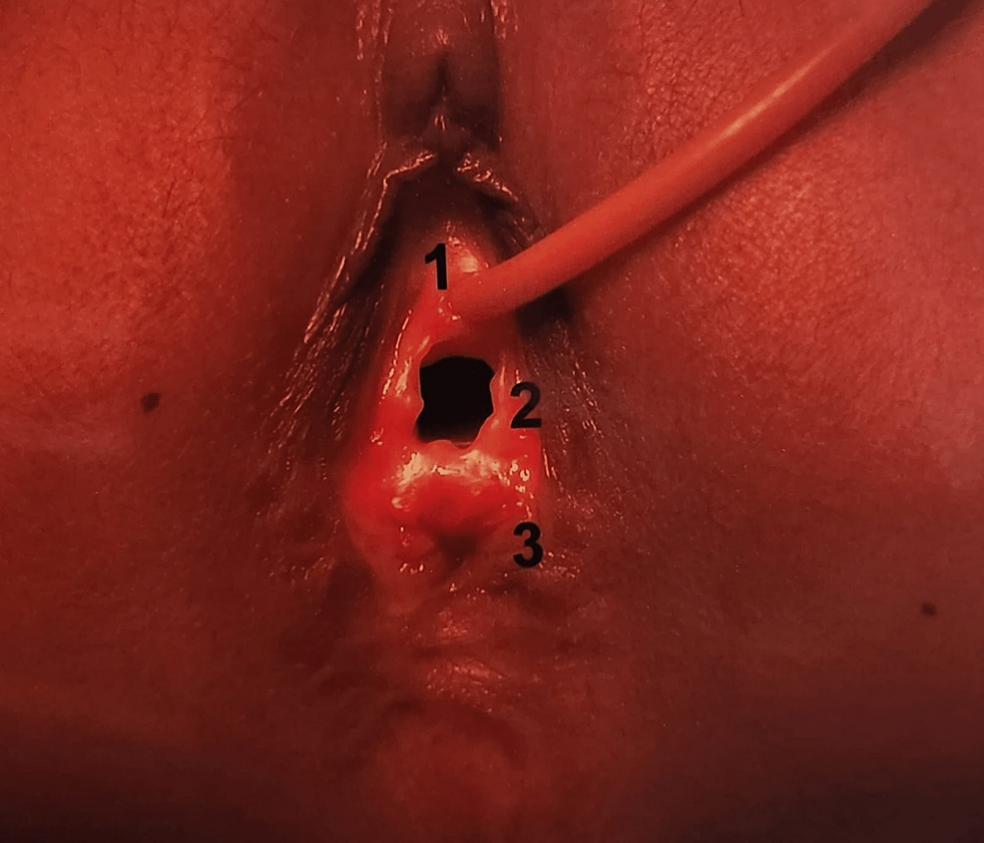
Anovestibular Fistula 1. Urethral opening 2. Vagina 3. Anus

Out of the 69 males in the present study, anorectal agenesis without fistula was seen in 21 (30.43%) cases (Table 3). In the male patients, rectoprostatic, rectobulbar, rectovesical, and anocutaneous fistula was present among 8 (11.59%), 9 (13.04%), 2 (2.89%), and 16 (23.18%) of cases (Table 3). Table 4 depicts the distribution of fistula in female patients. Out of 31 females in the present study, anorectal agenesis with anovestibular fistula was seen in 19 (61.29%) cases (Figure 1). Anorectal agenesis without fistula was observed in two (6.45%) cases, and anorectal agenesis with rectovestibular fistula was also seen in two (6.45%) cases (Table 4). However, a cloaca was seen in three cases (9.67%) and pouch colon in five cases (16.12%) (Table 4).

**Table 3:** Distribution of Fistula in Males

Type of Defect	Number (69)	Percentage
Anorectal Agenesis without Fistula	21	30.43
Anorectal Agenesis with Rectoprostatic Fistula	8	11.59
Anorectal Agenesis with Rectobulbar Fistula	9	13.04
Anorectal Agenesis with Rectovesical Fistula	2	2.89
Anocutaneous Fistula	16	23.18
Pouch Colon	13	18.84

 

**Table 4:** Distribution of Fistula in Females

Type of Defect	Number (31)	Percentage
Anorectal Agenesis without Fistula	2	6.45
Anorectal Agenesis with Rectovestibular Fistula	2	6.45
Anorectal Agenesis with Anovestibular Fistula	19	61.29
Ano Rectal Agenesis with Rectovaginal Fistula	0	0
Cloaca	3	9.67
Pouch Colon	5	16.12

**Figure 2:**
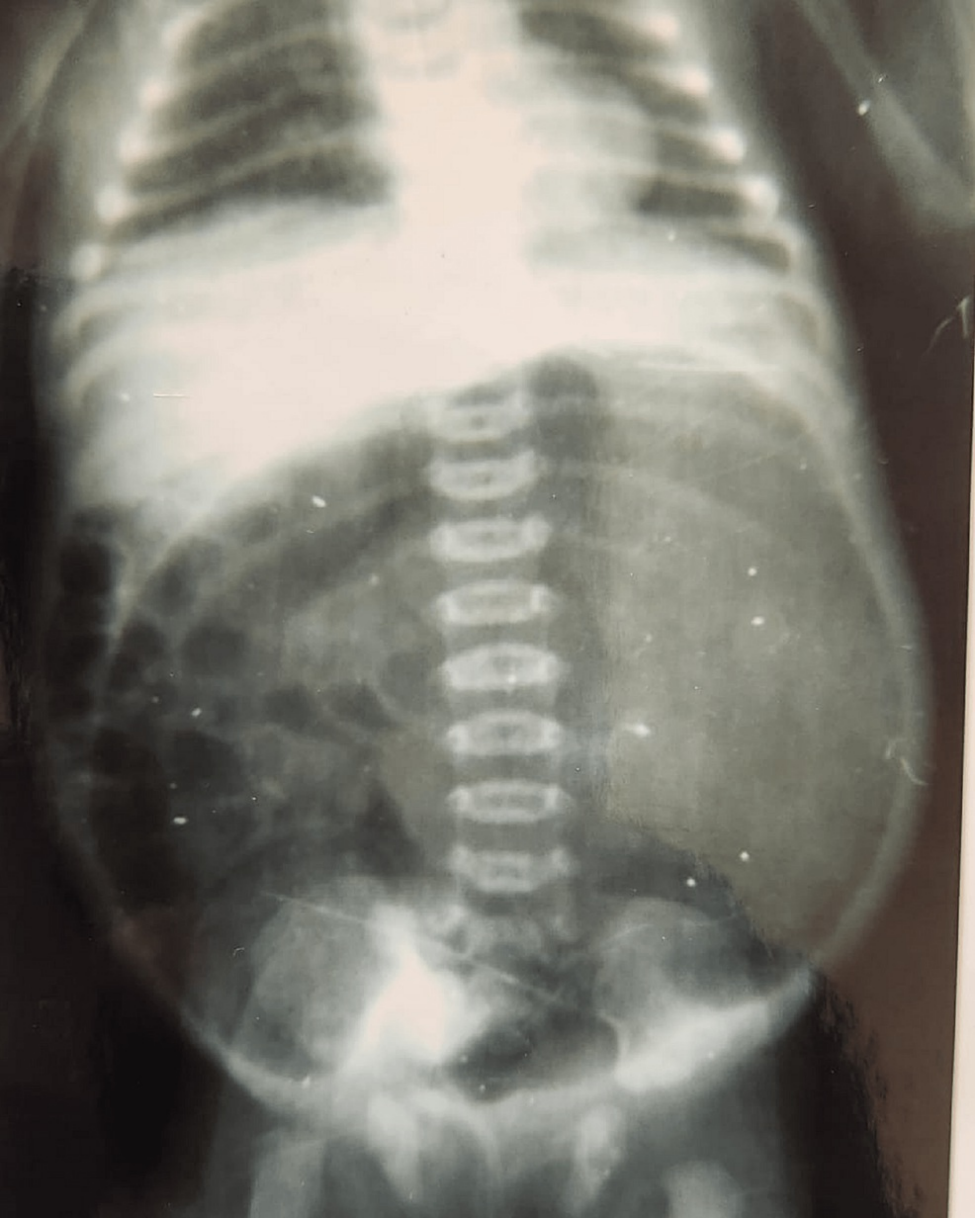
X-ray of the Abdomen With Pouch Colon

**Figure 3:**
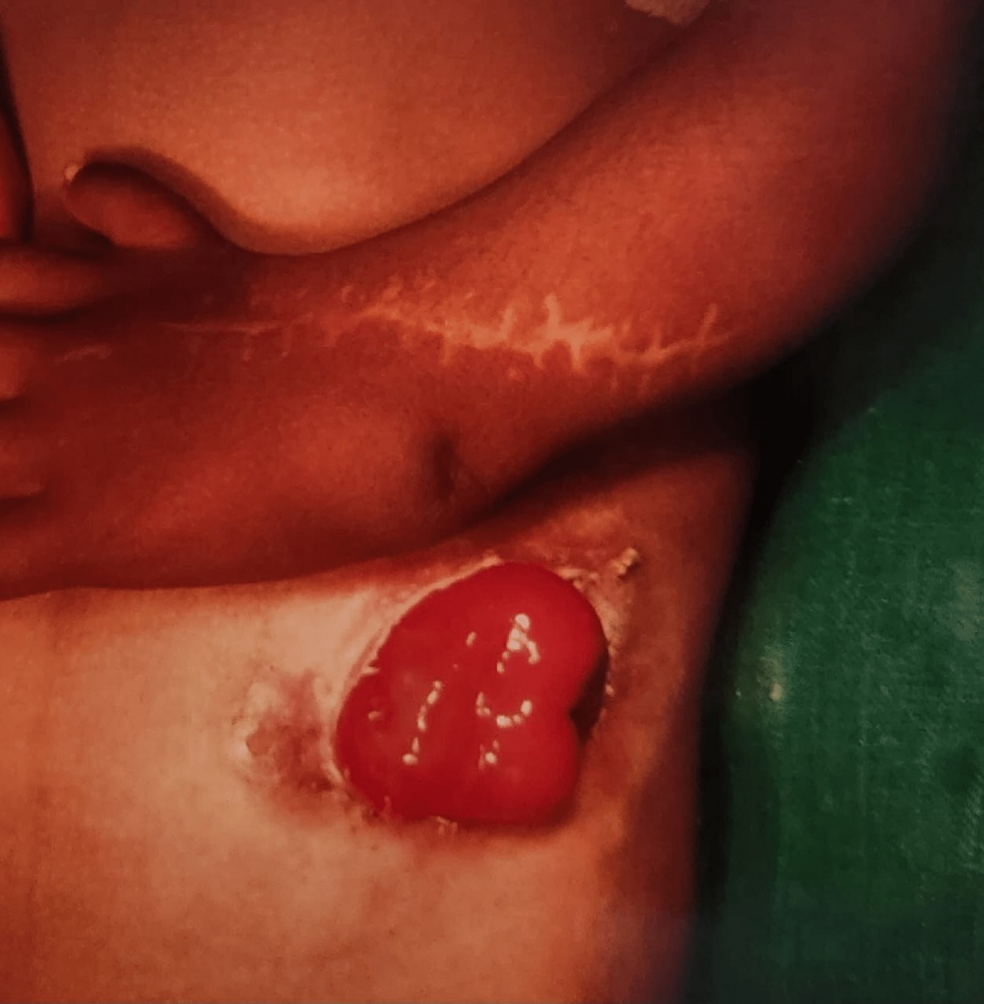
High ARM with Sigmoid Loop Colostomy ARM: Anorectal malformation

**Figure 4:**
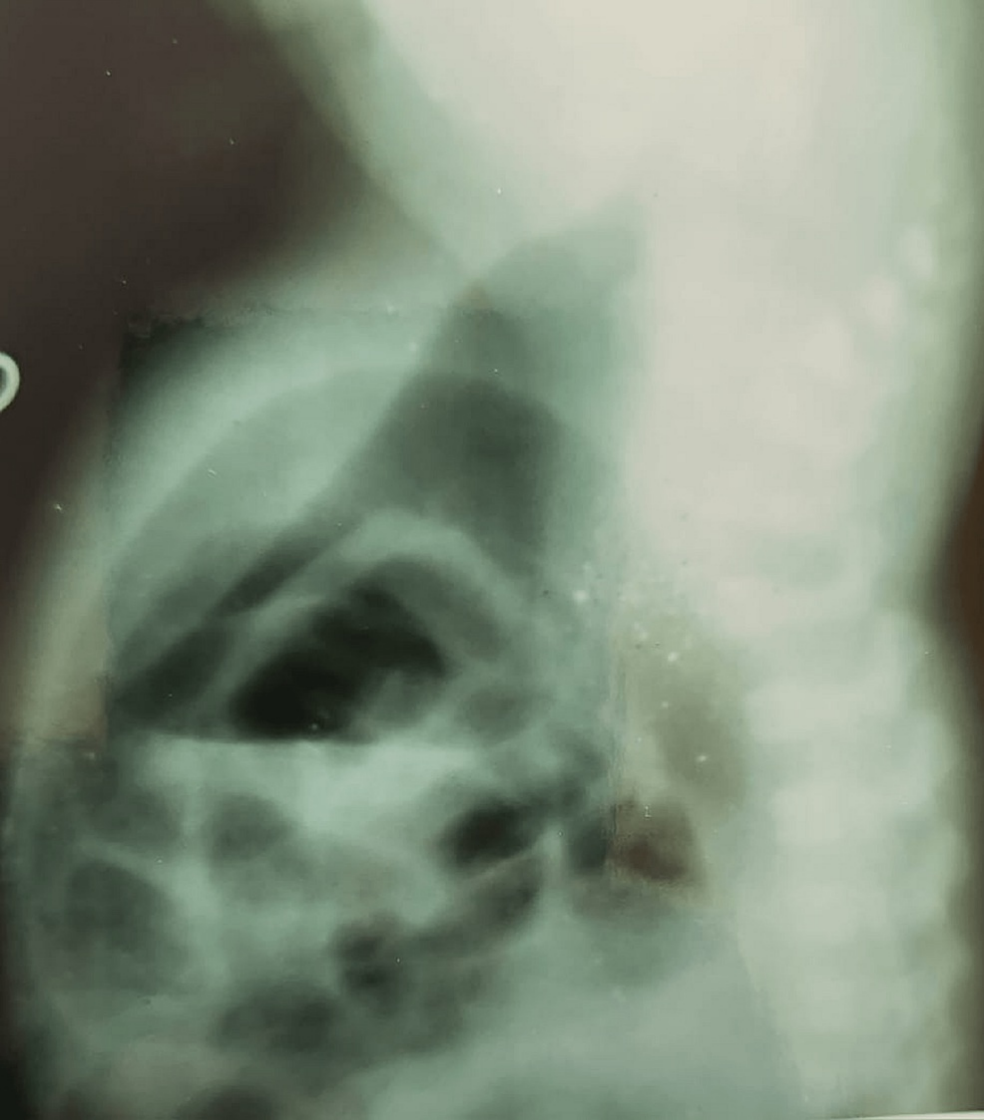
Invertogram Showing a High ARM ARM: Anorectal malformation

Table 5 depicts that 18 patients with ARMs had CPC (Figure 2). Out of these, males and females were 13 and 5, respectively. The incomplete/partial type III and IV CPC was present in 11 cases, whereas complete/total type I and II was found to be in seven cases (Table 5). Out of 63 cases of high ARMs, primary PSARP was done in two cases, remaining cases underwent staged procedures. Sigmoid, transverse, and descending colostomy were done in 27, 18, and 3 cases, respectively (Table 6) (Figures 3, 4). However, ileostomy and window colostomy were performed in six cases and one case, respectively. In 21 cases of PSARP, abdomen perineal pull-through was done in 15 cases (Table 6).

**Table 5:** Congenital Pouch Colon CPC: Congenital Pouch Colon Type 1 CPC: Normal colon is absent and ileum opens into pouch colon; Type 2 CPC: Ileum opens into a normal cecum which opens into pouch colon; Type 3 CPC: Normal ascending colon and transverse colon open into pouch colon; Type 4 CPC – Normal colon with rectosigmoid pouch.

Congenital Pouch Colon (CPC)	Number	Percentage
Total no. of CPC	18	18 (of total ARM)
Total No. of male CPC	13	72.22 (of CPC Patients)
Total No. of Female CPC	5	27.77 (of CPC patients)
Type of CPC		
Incomplete / Partial CPC (Type III & IV)	11 (Type III-5, IV-6)	61.11
Complete/total CPC (Type I & II)	7 (Type I-4, Type II-3)	38.88

 

**Table 6:** Operative Procedures Done in the High Type of Anorectal Malformation PSARP: Posterior sagittal anorectoplasty

Operative procedures		No. of cases
Initial Procedures Done	Sigmoid Colostomy	27
	Transverse Colostomy	18
	Descending Colostomy	3
	Ileostomy	6
	Window colostomy	1
	Total	55
Definite Procedure Done	PSARP	21
	Abdomen perineal Pull through	15
	Primary PSARP	2
	Total	38
Colostomy Closure		18

Table 7 depicts the various complications of colostomy. Local excoriation was observed in five (9%) patients, prolapse of the distal loop in eight patients (14.54%), and necrosis of stoma in two cases (3.63%) were observed, and one patient (1.82%) developed stenosis. Anemia and malnutrition were seen in eight cases (14.54%). In the early complications, significant bleeding and urethral injury were seen in one (2.63%) patient each (Table 8). However, among the late complications, anal stenosis, mucosal prolapse, and wound infection were seen in seven (18.42%), five (13.15%), and four (10.52%) patients, respectively. Neurogenic bladder and chronic anemia were seen in one and four patients, respectively. In the present study, following ASARP superficial wound infection and wound dehiscence were observed as early complications in three patients and one patient, respectively (Table 9). However, mucosal prolapse, anal stenosis, and constipation were seen in one patient each. 

**Table 7:** Complications of Colostomy

Complications	Number	Percentage
Local Excoriation	5	9.09
Prolapse of Distal Loop	8	14.54
Necrosis of Stoma	2	3.63
Stenosis	1	1.82
Anemia and Malnutrition	8	14.54

**Table 8:** Complications during and after the Definite Procedure for a High ARM ARM: Anorectal malformation

Complications		Number	Percentage
Early	Significant Bleeding	1	2.63
	Bladder Base Injury	0	0
	Urethral Injury	1	2.63
Late	Anal Stenosis	7	18.42
	Mucosal Prolapse	5	13.15
	Wound Infection	4	10.52
	Fecal Leak	0	0
	Neurogenic Bladder	1	2.63
	Chronic Anemia	4	10.52

**Table 9:** Complications after the Anterior Sagittal Anorectoplasty Technique

Complications		Number	Percentage
Early	Superficial Wound infection	3	23.07
	Wound Dehiscence	1	7.69
Late	Retraction of Anal Opening	0	0
	Mucosal Prolapse	1	7.69
	Anal Stenosis	1	7.69
	Constipation	1	7.69

## Discussion

In the present study out of 100 patients, 63 were of high type and 37 of a low type of anomaly. Out of 63 patients of high variety, 53 (84.12%) were males and 10 (15.87%) were females, and out of 37 patients of low type, 16 (43.24%) were males and 21 (56.75%) were females. Thus, male children have a higher chance of having a high variety of lesions than female children [5,6,7]. In the present study, we observed that children with a high type of anomaly have an increased incidence of associated anomalies. 

**Figure 5:**
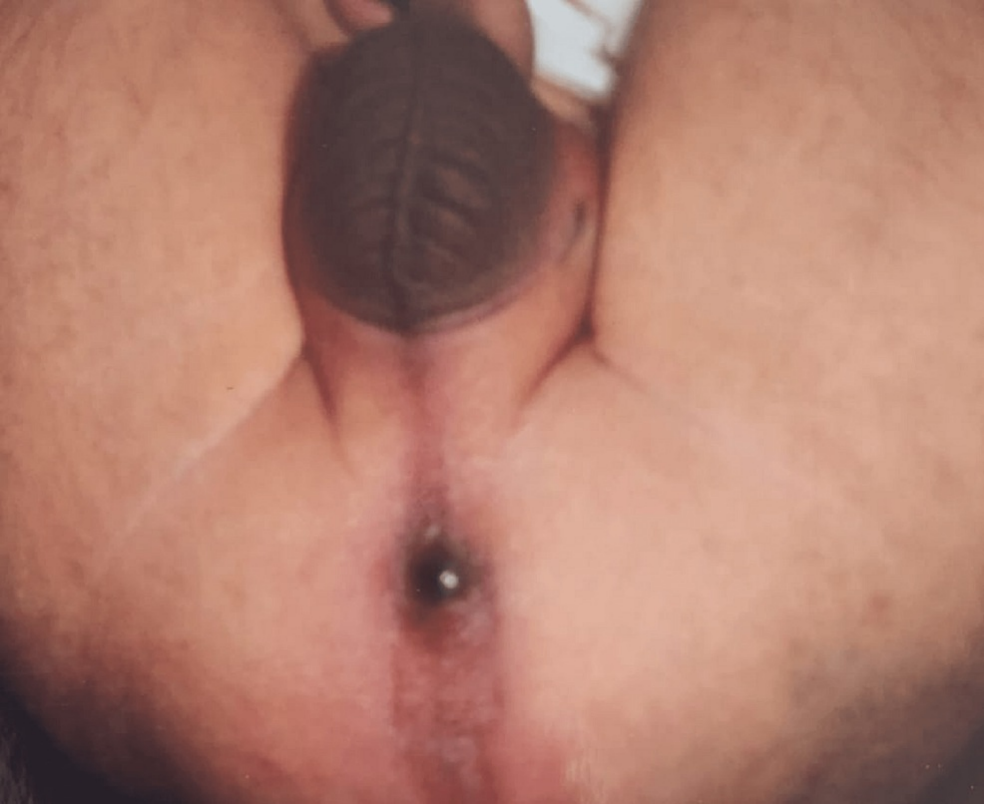
Low ARM with Meconium ARM: Anorectal malformation

The high type of ARMs was most prevalent, which is in consonance with global experience [8]. In the present study, out of 63 cases of high ARMs, PSARP was done in two cases, and the rest of the cases underwent staged procedures. Sigmoid colostomy was done in 27 cases, transverse colostomy in 18 cases, descending colostomy in three cases, ileostomy in six cases, and window colostomy in one case. These were followed by PSARP in 21, and abdomen perineal pull-through in 15 cases. Colostomy closure was done in 18 cases. The rest of the colostomy closures was done after completion of the study period. Out of 37 cases of low ARMs, anoplasty was done in 37 patients and ASARP in 13 patients (Figure 5).

Colostomy has long been a common preliminary treatment in the surgical management of many congenital and acquired conditions of the gastrointestinal tract among children, and many are done on an emergency basis [9,10]. Although three-stage surgeries have been in practice in the management of ARMs and have been known to be most effective in preventing complications, recently many authors have opted for primary PSARP [4,11,12]. In our study, as per Kelly’s Score, 35 patients were assessed for continence, 20 (57.14%) had good, 14 (40%) had fair, and 1 (2.86%) had poor continence. Others also reported similar outcomes with “good bowel motion” or fecal continence in 62.5-90.1% following surgery for children with ARMs.

In addition, the prognosis for continence tends to be better in children who had surgical interventions earlier than later [13]. In the present study out of 100 cases, 20 patients (18 High, 2 low type) died due to various causes like cardiac anomalies in three patients, tracheoesophageal anomalies in four patients, and renal anomalies in four patients. So, in the study 11/20 (55%) died due to associated anomalies. Other than these three patients died due to perforation peritonitis, two patients due to septicemia, and 3 due to prematurity, low birth weight, and hypothermia. One patient died postoperatively due to anesthesia complications. Overall, five patients died without operation due to serious anomalies, whereas in other studies, the overall mortality in children with the ARM range from 4.3 to 31.0%. The mortality rate is higher in children with associated malformations than those with “higher” malformations and in the neonatal period [14-20].

In the present study, following staged ASARP superficial wound infection and wound dehiscence were observed as early complications in three patients and one patient, respectively. However mucosal prolapse, anal stenosis, and constipation were seen in one patient each. Gupta et al. did a similar study among female patients who had undergone staged ASARP having low-type ARMs and found similar results [21]. Jerry et al. conducted an observational study of neonates with gastrointestinal abnormalities including anorectal malformation and suggested that early management of associated anomalies is essential for the good prognosis of these patients [22]. 

Baxter et al. conducted extensive research on bowel management strategies in children with ARMs [23]. De-identified clinical data on ARM patients were sent to a consolidated patient registry by seven American hospitals. The Pediatric Colorectal and Pelvic Learning Consortium proposed the first-ever report on bowel management program (BMP) strategies for patients with ARMs. In the report, individual patient characteristics are explored for their impact on bowel management strategy utilization.

Peña et al. studied the recent advances in the management of ARMs [24]. Pediatric surgeons continue to face a struggle with anorectal abnormalities. Many of these children have issues with fecal incontinence, urine incontinence, and inadequate sexual development. Among all patients, 75% of cases had voluntary bowel movements. Out of which, 50% still occasionally had fecal incontinence. Therefore, about 37.5% of all cases were considered totally continent. The most common sequela was constipation. However, after the repair of the cloaca, urinary incontinence was more common. Urinary incontinence was rarely observed among male patients. The bowel management program was helpful in improving the quality of life in 25% of all cases who suffered from fecal incontinence. An operation called “continent appendicostomy” further improved their quality of life.

As this is a cross-sectional study, a cause-effect relationship cannot be ascertained. Moreover, it is a non-randomized study, so the results cannot be generalized to the entire population. A multi-institutional and multicentric study can be done to further strengthen the results. The study carries the limitations of a small sample size. A better outcome can be predicted if the study sample size is larger. Moreover, the study duration was only three years. A longer duration study may better predict the treatment outcome of the patients.

## Conclusions

In India, ARMs account for a major part of the workload of pediatric surgeons. In spite of the progress made in the field of neonatal surgery, a high volume of patients and associated malformations compounded in a resource-limited country negatively influence the outcome of ARM patients. The outcome of surgery is dependent on the specific type of malformation, but the results are better when intervention is done early. In this study, the mortality was 20%, and high mortality was seen among neonates who had associated anomalies and delayed presentations. All newborns presenting with ARMs should be screened for associated anomalies to avoid morbidity and mortality.
